# Dendritic Compartmentalization of Learning-Related Plasticity

**DOI:** 10.1523/ENEURO.0060-22.2022

**Published:** 2022-06-22

**Authors:** Luca Godenzini, Adam S. Shai, Lucy M. Palmer

**Affiliations:** 1 Florey Institute of Neuroscience and Mental Health, University of Melbourne, Victoria 3052, Australia; 2 CNC Program, Stanford University, Stanford, CA 94305

**Keywords:** compartmentalization, dendrites, fear learning, two-photon calcium imaging, whole-cell patch clamp in vivo

## Abstract

The dendrites of cortical pyramidal neurons receive synaptic inputs from different pathways that are organized according to their laminar target. This architectural scheme provides cortical neurons with a spatial mechanism to separate information, which may support neural flexibility required during learning. Here, we investigated layer-specific plasticity of sensory encoding following learning by recording from two different dendritic compartments, tuft and basal dendrites, of layer 2/3 (L2/3) pyramidal neurons in the auditory cortex of mice. Following auditory fear conditioning, auditory-evoked Ca^2+^ responses were enhanced in tuft, but not basal, dendrites leading to increased somatic action potential output. This is in direct contrast to the long held (and debated) hypothesis that, despite extensive dendritic arbors, neurons function as a simple one-compartment model. Two computational models of varying complexity based on the experimental data illustrated that this learning-related increase of auditory responses in tuft dendrites can account for the changes in somatic output. Taken together, we illustrate that neurons do not function as a single compartment, and dendritic compartmentalization of learning-related plasticity may act to increase the computational power of pyramidal neurons.

## Visual Abstract

**Figure:**
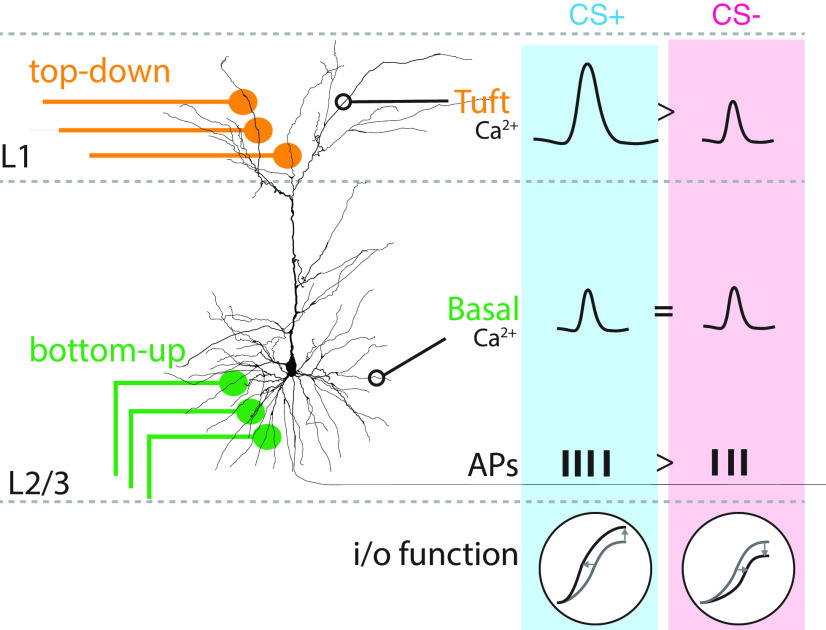

## Significance Statement

This study directly investigates whether information processing in neurons is compartmentalized within different dendritic regions. Our findings shed light on the learning-related changes that occur in dendritic compartments residing in different layers of the cortex (i.e., tuft and basal dendrites), illustrating that layer 2/3 (L2/3) pyramidal neurons are able to compartmentalize learning signals which leads to enhanced somatic output. The compartmentalization of experience-dependent plasticity supports flexible sensory processing and may increase the computational power of single pyramidal neurons. In addition, it highlights the special role of tuft dendrites, the target of top-down inputs, in modulating sensory encoding following fear learning.

## Introduction

Cortical pyramidal neurons lay at the heart of sensory processing. As the target of synaptic input, cortical dendrites encode sensory stimuli and perception (Xu et al., 2012; Palmer, 2014; Takahashi et al., 2016, 2020; Ranganathan et al., 2018) and ultimately influence neuronal firing (Stuart et al., 1997; Helmchen et al., 1999; Larkum et al., 1999; Golding et al., 2002; Losonczy et al., 2008; Lavzin et al., 2012; Harnett et al., 2013; Smith et al., 2013; Palmer et al., 2014; Manita et al., 2015). Despite their extensive and distinctive morphology, dendrites have historically been treated as a single entity, acting as a single compartment to sum synaptic input (Rall, 1962; Mainen and Sejnowski, 1996; Segev and Rall, 1998; Häusser and Mel, 2003). However, within a single neuron, different information pathways target different regions of the dendritic tree. Basal dendrites, which emanate from the soma of pyramidal neurons, largely receive sensory input and are thought to play an important role in the processing of sensory feedforward (or bottom-up) information (Bannister, 2005; Petreanu et al., 2009). Conversely, distal tuft dendrites, residing in the upper layers of the cortex, largely receive feedback (or top-down) input from higher order areas (Petreanu et al., 2009) and are thought to play an important role in the modulation of sensory encoding (Cauller, 1995; Petreanu et al., 2012; Larkum, 2013; Makino and Komiyama, 2015; Manita et al., 2015; Roth et al., 2016; Marques et al., 2018; Keller et al., 2020). Since they are morphologically separated, the distal tuft and basal dendrites of cortical pyramidal neurons provides individual neurons with a cellular mechanism to simultaneously and independently process different information pathways (Koch et al., 1982; Häusser and Mel, 2003; Poirazi et al., 2003; Beaulieu-Laroche et al., 2018). By providing multiple independent integrative units, dendritic compartmentalization in cortical neurons may ultimately act to increase the computational power of individual neurons and support the neural flexibility required during learning (Koch et al., 1982; Häusser and Mel, 2003; Poirazi et al., 2003; Beaulieu-Laroche et al., 2018).

In sensory cortices, learning influences local circuits (Makino and Komiyama, 2015; Audette et al., 2019) leading to local plasticity (Bakin and Weinberger, 1990; Edeline et al., 1993; Weinberger, 1993; Weinberger et al., 1993) and altered network activity (Gdalyahu et al., 2012; Kass et al., 2013; Makino and Komiyama, 2015; Lacefield et al., 2019). Since experience-dependent plasticity can occur at the level of a single dendrite (Holthoff et al., 2006; Makino and Malinow, 2011; Cichon and Gan, 2015; Brandalise et al., 2016; Sandler et al., 2016; Bittner et al., 2017), cortical dendrites are ideal candidates for driving the specific and dynamic changes in sensory processing that occur during learning. Here, we tested this by investigating whether auditory fear learning influences the processing of auditory information in the dendrites of cortical pyramidal neurons. By focusing on both the distal tuft and basal dendrites of layer 2/3 (L2/3) pyramidal neurons, which reside in different cortical layers, we assessed whether learning causes different dendritic compartments to undergo similar plasticity during sensory processing, or whether plasticity is compartmentalized in the physically separate dendrites. Using two-photon Ca^2+^ imaging in the auditory cortex of anaesthetized mice, we reveal a learning-related increase in auditory-evoked Ca^2+^ responses in tuft, but not basal, dendrites. Fear learning also enhanced the somatic action potential output, despite no change in the auditory-evoked subthreshold voltage response. Computational models based on the experimental data further showed that our results are consistent with tuft dendrites playing a primary role in driving the enhanced somatic output following learning. Taken together, the specific learning-related plasticity in tuft, but not basal, dendrites suggests that plasticity can be compartmentalized within cortical pyramidal neurons, leading to increased somatic output.

## Materials and Methods

All experiments were conducted in strict accordance with the Code of Practice for the Care and Use of Animals for Scientific Purposes (National Health and Medical Research Council, Australia) and guidelines given by the veterinary office at the Florey Institute of Neuroscience and Mental Health.

### Auditory fear conditioning

Mice [C57BL/6; postnatal day (P)42–P70, females] were exposed to an auditory fear conditioning protocol. Mice were placed in fear conditioning chambers (MedAssociates) for 2 min of habituation before trains of auditory tones were presented at 1 Hz (pure tones: 5 or 15 kHz; 5 × 500-ms duration, 500-ms Inter Stimulus Interval (ISI)). The auditory trains were presented either with (CS+) or without (CS–) a 0.6-mA footshock for six times each (5 s each), in a block design (CS+ following the CS– block). The onset of the footshock coincided with the onset of the last tone in the train of tones. After 24 h, CS+ and CS– were presented again in a different context (eight repetitions each, duration 10 s) and freezing behavior were recorded and compared. Freezing scores were automatically measured by the software (MedAssociates) as the percentage of freezing during auditory stimulus presentation, corrected with the baseline values (first 2 min of habituation). Mice that failed to discriminate between the CS– and CS+ (<30% difference in freezing) were excluded from additional experiments and recordings.

### Virus injections

Mice (C57BL/6; P30–P42) were anaesthetized with isoflurane (3% in 0.75 l/min O_2_) and body temperature was maintained at 36–37°C. Eye ointment was applied to prevent dehydration. Meloxicam (1–3 mg/kg, Ilium) was intraperitonially injected at the beginning of the surgery. The skin was disinfected with ethanol 70% and betadine and a small slit was made in the skin to expose the skull. A small craniotomy (<0.5 mm) was made over the left auditory cortex (2.5 mm posterior to bregma and 4.5 mm lateral from midline) and the dura was left intact. To obtain sparse labeling of L2/3 pyramidal neurons, a mix of the Cre-dependent Ca^2+^ indicator, GCaMP6f (AAV1.Syn.Flex.GCaMP6f.WPRE.SV40, UPenn) and diluted Cre (1:6000; AAV1.hSyn.Cre.WPRE.hGH, UPenn) was injected at a dorsoventral distance of 450 μm with a microcapillary pipette. Finally, the skin was sutured and the mouse put back into their cage for a minimum of three weeks to allow expression of GCaMP6f, before two photon imaging.

### Auditory stimulation

During two-photon Ca^2+^ imaging and whole-cell patch clamp voltage recordings, auditory stimulation (pure tones: 5 or 15 kHz; 5 × 500-ms duration, 500-ms ISI; 20 trials each) was provided through a speaker (8 Ω, 5 W; digikey) positioned on the right side of the mouse ∼8–10 cm from the contralateral ear. The auditory stimuli were custom made and delivered with an Arduino processing board at ∼70 dB.

### Two-photon Ca^2+^ imaging and image analysis

Mice previously transfected with GCaMP6f were initially anaesthetized with isoflurane (3% in 0.75 l/min O_2_) before urethane anesthesia (1.6 g/kg, i.p.; Sigma) was administered. After removing the skin and cleaning the skull with NaOH (3%), a craniotomy was performed (3 mm diameter) over the virus injection site in the auditory cortex (left hemisphere). A circular coverslip (3 mm diameter, size #1) was placed over the craniotomy and sealed with glue. A custom-made metal head-bar (0.5 g) was attached to the skull using dental cement (paladur, Heraeus). For dendritic imaging, GCaMP6f was excited at 940 nm (∼30 mW at the back aperture) with a titanium sapphire laser (140-fs pulse width; SpectraPhysics MaiTai Deepsee) and imaged on a Sutter MoM through a 16× Nikon objective (0.8 NA). Emitted light was passed through a dichroic filter (565dcxr, Chroma Technology) and short-pass filtered (ET525/70-2p, Chroma Technology) before being detected by a GaAsP photomultiplier tube (Hamamatsu). Images were acquired at a frequency of 30 Hz (512 × 512 pixels) using ScanImage software. Ca^2+^ activity was recorded from visually identified tuft and basal dendrites of L2/3 pyramidal neurons within the auditory cortex at a depth of 30–80 and 150–250 μm from the pia surface, respectively. To ensure all reported dendrites are from L2/3 pyramidal neurons, we visually followed tuft dendrites to the soma of origin and performed Z-stacks in all experiments. Horizontal and vertical drifts of imaging frames because of animal motion were corrected by registering each frame to a reference image based on whole-frame cross-correlation. The reference image was generated by averaging frames for a given field of view (FOV) in which motion drifts were minimal (>15 pixels). Regions of interest (ROIs) were selected using the SD of all frames in randomly selected trials (five to eight trials, ∼2000–4000 frames, spanning the entire imaging session) and manually drawn using the freehand tool in ImageJ. ROIs were selected so that each ROI represented a single dendrite. For display purposes only, images were gamma corrected.

### Ca^2+^ analysis

All Ca^2+^ signal processing was performed with custom written MATLAB scripts. Fluorescence traces are expressed as relative fluorescence changes, *ΔF/F* = *(F – F_0_)/F_0_*. Ca^2+^ responses were smoothed using a Savitzky–Golay filter (second order polynomial and seven sample window). Baseline fluorescence (F_0_) for each ROI was calculated from the average baseline florescence intensity during 5 s before the stimulus onset of each trial. For each dendrite, the Ca^2+^ traces for all trials were averaged together to generate a trial-averaged Ca^2+^ response. Evoked Ca^2+^ responses were detected if they were greater than 3× the SD of the baseline (5 s before the auditory stimulus onset). Peak amplitudes of the trial-averaged Ca^2+^ responses were measured from when the fluorescence trace crossed the 3x baseline SD threshold value. To calculate the integral of the Ca^2+^ response, trapezoidal numerical integration was performed on the trial-averaged Ca^2+^ response normalized to the maximum amplitude of the evoked response. Unless otherwise stated, data are presented as the mean response and error bars represent the SEM. 3D reconstruction and tracing of tuft dendrites was performed using the NeuTube software. Correlation coefficients were measured as the total number of Ca^2+^ transients occurring within sister dendritic branches during a trial divided by the total number of sister branches recorded for a given neuron.

### Whole-cell recordings under anesthesia

Mice (C57BL/6; P42–P63) were initially anaesthetized with isoflurane (3% in 0.75 l/min O_2_) before urethane anesthesia (1.6 g/kg, i.p.; Sigma) was administered. Anesthesia was monitored throughout the experiment, and a top-up dose of 10% of the initial urethane dose was administered when necessary. Body temperature was maintained at 36–37°C. Lidocaine (20 mg/ml, Ilium) was injected around the surgical site on the scalp and the head was stabilized in a stereotaxic frame by a head-plate attached to the skull with dental cement (paladur, Heraeus). A craniotomy was performed over the left auditory cortex (∼1.5 × 1.5 mm^2^), centered at −2.5 mm from bregma and 4.5–5 mm lateral from midline. The dura was surgically removed and the brain was constantly bathed with normal rat ringer (135 mm NaCl, 5.4 mm KCl, 1.8 mm CaCl_2_, 1 mm MgCl_2_, 5 mm HEPES) throughout the experiment. Whole-cell *in vivo* patch clamp recordings were performed using a patch pipette (resistance 6–9 MΩ) filled with an intracellular solution containing 115 mm potassium gluconate, 20 mm KCl, 10 mm sodium phosphocreatine, 10 mm HEPES, 4 mm Mg-ATP, 0.3 mm Na-GTP, adjusted to pH 7.3–7.4 with KOH. The patch pipette was inserted into the brain at an angle of 30°- 40° relative to the cortical surface, to a depth of ∼200 μm (to target L2/3 neurons). The pipette was then advanced in steps of 1 μm (to a maximum distance of 300 μm in the hypotenuse trajectory) until a neuron was encountered. Whole-cell voltage recordings were performed from the soma using Dagan BVC-700A amplifiers and were filtered at 10 kHz. Once a whole-cell recording was obtained, the voltage response to incremental current steps (50 pA; 800 ms) was recorded to characterize the neuron. In a subset of neurons which had a low rate of action potential firing, positive holding current was applied to the neuron via the patch pipette (∼50 pA) to provide additional depolarization to lower the threshold for action potential generation. Custom-written Igor software was used for the acquisition and of whole-cell recordings. In a subset of experiments, 5-(and-6)-Tetramethylrhodamine biocytin (biocytin-TMR, ThermoFisher) was added to the patch pipette and released extracellularly after the recordings to identify the auditory cortex *post hoc*. Brains were fixed with paraformaldehyde (PFA; 4%), sliced on a vibratome (200 μm; Leica) and fluorescence imaging was performed to confirm the location of the recordings.

### Data analysis

For whole-cell recordings, the evoked subthreshold response was analyzed as the integral of the evoked voltage envelope (for each tone) using a custom written script in Igor. The firing rate was measured as the number of action potential detected in a 500-ms window from each tone onset divided by the number of trials.

### Drug application

During *in vivo* whole-cell patch clamp recordings, APV (200 μm diluted 20× in ringer; Tocris) was topically applied over the craniotomy and current clamp recordings were performed.

### Statistical analysis

Data were tested for normality and parametric or nonparametric test were used accordingly using the software GraphPad Prism (version 9.0.0). For paired data, Wilcoxon matched-pairs signed-rank test was used for statistical analysis. For unpaired data, Mann–Whitney test was used for statistical analysis. Statistical tests are reported in each figure caption. All data are reported as mean value ± SEM.

### Computational model

A multicompartmental computational model of a L2/3 pyramidal neuron was simulated based on previous work (Palmer et al., 2014). Details of the parameters of the model are given in Extended Data Figure 4-1. The model and code to run all simulations will be available on ModelDB (https://senselab.med.yale.edu/modeldb/) at the time of publication. Intrinsic membrane mechanisms were taken from the Hay et al. (2011) layer 5b pyramidal model and consisted of 10 active conductances, internal Ca^2+^ dynamics, and passive conductances. Values were chosen by starting with the biophysics of the Hay and colleagues model and modifying them to better fit experimental data for L2/3 pyramidal neurons. The main changes were decreases in I_h_ and Ca^2+^ HVA and LVA channel conductances and increases in the two S_k_ channel conductances (see Extended Data Fig. 4-1 for details).

**Figure 1.**
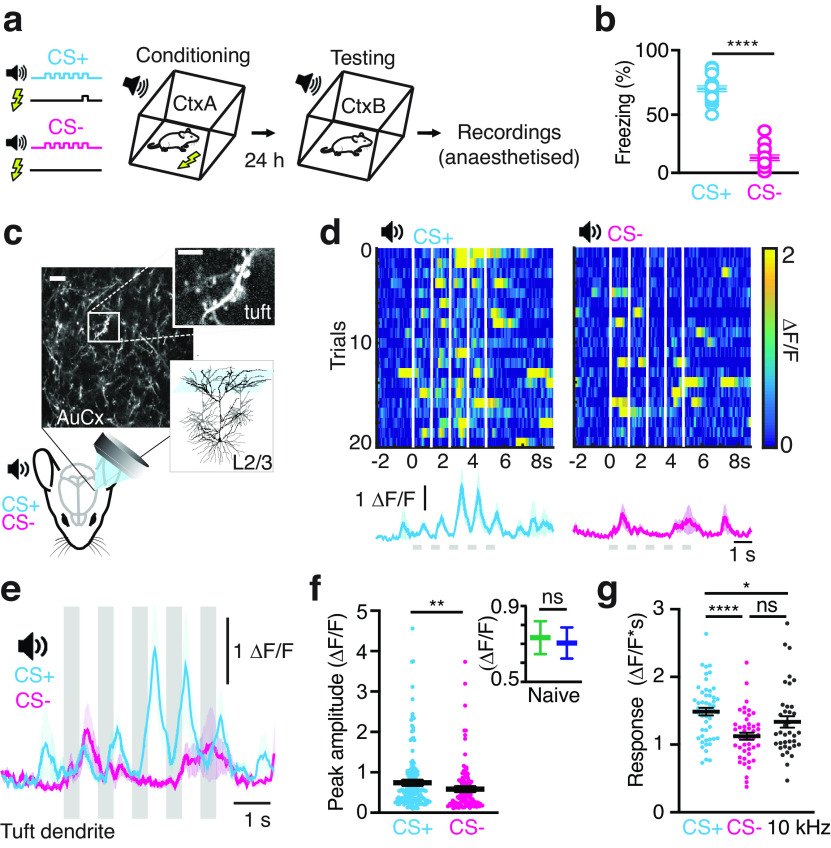
Auditory-evoked Ca^2+^ responses to CS+ are enhanced in tuft dendrites. ***a***, Schematic of the experimental design and of the auditory fear conditioning protocol. ***b***, Freezing scores to CS+ and CS– 24 h after conditioning (*n* = 20 mice; paired *t* test). ***c***, Schematic of the experimental design. Example field of view (FOV) of tuft dendrites from L2/3 pyramidal neurons in the auditory cortex expressing GCaMP6f. Scale bar: 10 μm; 5 μm (inset). ***d***, Top, Heatmaps of Ca^2+^ transients in response to CS+ (left) and CS– (right) in an example tuft dendrite. White bars indicate timing of the tones (5 × 500 ms). Bottom, Trial-averaged Ca^2+^ responses to CS+ (cyan) and CS– (magenta). Bold line, Average response; light shading, SEM. ***e***, Overlay of the trial-averaged Ca^2+^ responses to CS+ (cyan) and CS– (magenta) from the same tuft dendrite shown in ***d***. Gray bars show the tones in the auditory stimuli. ***f***, Peak amplitude of the trial-averaged Ca^2+^ responses to CS+ (0.74 ± 0.05 ΔF/F) and CS– (0.58 ± 0.05 ΔF/F). Mann–Whitney test. Inset, Peak amplitude of the trial-averaged Ca^2+^ responses in naive mice were not different (see also Extended Data Fig. 1-1). ***g***, Integral of the trial-averaged Ca^2+^ responses to CS+ (1.48 ± 0.05 ΔF/F*s), CS– (1.12 ± 0.05 ΔF/F*s), and a reference stimulus (10 kHz; 1.33 ± 0.08 ΔF/F*s; Extended Data Fig. 1-2). Mann–Whitney test. All data represented as mean ± SEM. ns: *p* > 0.05, * *p* < 0.05, ** *p* < 0.01, **** *p* < 0.0001. In a subset of experiments, tuft dendrites were traced to their soma of origin to measure the correlation of activity across dendritic branches of the same neuron (Extended Data Fig. 1-3).

10.1523/ENEURO.0060-22.2022.f4-1 Extended Data Figure 4-1 Details of the L2/3 pyramidal neuron computational model. Please see materials section for further explanation. ***a***, The reconstruction of the pyramidal neuron used in the model, with tuft and basal dendrites colored in red and orange, respectively. ***b***, Examples of the location of background (left), basal (middle), and tuft (right), synapses used in the simulations. Each synapse is a combination of AMPA and NMDA conductances, and it is shown as a red dot. ***c***, Table of conductances values used in the simulation. For the Ih channel in the tuft, conductance was a function of the distance from the soma, –0.8696 + 2.087 exp(x/323), where x was distance from the soma in microns. ***d***, The somatic voltage response to 2-s-long current steps into the soma, from –1.0- to 0.7-nA steps of 0.1 nA. ***e***, example action potential evoked in response to a current injection at the soma of 3 nA for 2 ms. ***f***, The spike frequency output as a function of DC current steps into the soma. ***g***, The steady state somatic voltage as a function of DC current into the soma. ***h***, A total of 30 examples runs of only background synaptic inputs. Download Figure 4-1, EPS file.

To model the effects of fear learning, excitatory synaptic input was modelled by a voltage dependent NMDA and voltage independent AMPA conductance at each synapse, using a NEURON mechanism created by Alon Polsky on http://senselab.med.yale.edu/ and published previously (Larkum et al., 2009). The NMDA conductance was voltage dependent and given by g_NMDA_ = g_MAX_(exp(-t/70) -exp(-t/3))/(1 + 0.3 exp(−0.08v)), and AMPA conductances were modeled with an instantaneous rise time and decay time constant of 0.5 ms (Larkum et al., 2009). Each synapse had a maximum NMDA to maximum AMPA conductance ratio of 1:1. 75 background synapses were randomly distributed across the neuron and were given maximum conductances of 1 nS. Each background synapse was randomly activated between 10 and 100 Hz. To model stimulus-evoked synaptic input, top-down and bottom-up NMDA/AMPA synapses were distributed with uniform distribution across the tuft and basal dendrites. Each of the sensory inputs fired once at a randomly selected time during the stimulus window of 500 ms. To compare increasing tuft versus basal inputs on the model firing, we simulated 200 basal (or tuft) stimulus-evoked synapses with increasing tuft (or basal) stimulus-evoked synaptic inputs, from 200–300 synapses. A total of 4730 simulations were run, randomly selecting the locations and timing of each synapses for each simulation.

### Reduced model

To simulate auditory inputs onto a L2/3 pyramidal neurons in a model with fewer parameters, we adopted a composite sigmoid model, previously used to model the interaction of tuft and basal compartments of a L5 pyramidal neuron. This model has considerably fewer parameters, all of which are easily interpretable; they describe two tuft sigmoids which control the threshold and maximum of a basal sigmoid which converts basal input into a firing rate. The form this model took was the following:

$$M\left(t\right)=a_{1}+\frac{a_{2}}{1+e^{-\frac{t-a_{3}}{a_{4}}}}$$

$$T\left(t\right)=b_{1}+\frac{b_{2}}{1+e^{-\frac{t-b_{3}}{b_{4}}}}$$

$$F\left(b,t\right)=c_{1}+\frac{M(t)}{1+e^{-(b-T\left(t\right))}}$$

The independent variables *b* and *t* are the basal and tuft inputs, the functions *M(t)* and *T(t)* are each sigmoids parametrized by the parameters *a* and *b*, and control the maximum and threshold values of a third sigmoid, *F(b,t)* that describes the transformation of basal input into output frequency. The parameter *c_1_* describes the baseline firing rate. We binned the 500-ms auditory period into 10 50-ms periods. To find the parameters for our simulations that might fit the multicompartmental data, we employed a genetic algorithm, using the absolute difference between the curves in Figure 4*c*,*f* as a loss function. For each simulation we assigned all tuft and basal inputs times by sampling from a uniform distribution from 0 to 500 ms, and then binned each synapse into the 10 time periods of the simulation. For every time period, we ran the inputs through the composite sigmoid model to get a Poisson probability, and then sampled from that distribution to get a number of spikes in that time bin. Doing that for every bin gave us a total spike count, which we then converted into an output frequency, to get the plot in Figure 4*f*. The code for the reduced model and code for fitting using the genetic algorithm are available online at https://github.com/adamimos/FearfulDendrites.

## Results

### Auditory-evoked responses to CS+ are enhanced in tuft dendrites

To assess the influence of learning on dendritic encoding of sensory information, mice were exposed to an auditory fear conditioning protocol that consisted of trains of pure tones (5 × 500 ms, 5 or 15 kHz) presented with (CS+) or without (CS–) a mild footshock (Yang et al., 2016; Fig. 1*a*). The auditory stimuli (5 or 15 kHz) were counterbalanced and randomly allocated as either CS+ or CS–. Behavioral freezing responses to the auditory stimuli were tested the day following conditioning. Conditioned mice discriminated between the two stimuli, with CS+ reliably evoking more freezing compared with CS– (68.5 ± 2.3% vs 12.3 ± 2.1%; *n* = 20 mice; *p* = 0.0001; Fig. 1*b*). To determine how the processing of sensory information in cortical dendrites was influenced by fear learning, we performed two-photon calcium imaging in the dendrites of L2/3 pyramidal neurons within the auditory cortex of urethane anaesthetized mice sparsely transfected with the genetic Ca^2+^ indicator GCaMP6f. Since learning involves feedback information within the upper layers of sensory cortices (Makino and Komiyama, 2015; Doron et al., 2020), we first investigated whether sensory information at the dendritic site of feedback input in pyramidal neurons, tuft dendrites, is altered by auditory fear conditioning. Following the fear conditioning protocol, auditory-evoked Ca^2+^ activity was recorded in the tuft dendrites of L2/3 pyramidal neurons (<80 μm below pia; Fig. 1*c*,*d*). Overall, a similar number of tuft dendrites responded to the auditory stimuli presented during fear conditioning (CS+: 20% and CS–: 18%). However, in these dendrites, the peak of the average Ca^2+^ response evoked during CS+ was significantly larger than CS– (0.74 ± 0.05 vs 0.58 ± 0.05 ΔF/F; *n* = 52/46 dendrites, 9 mice; *p* = 0.005; Fig. 1*e*,*f*). In contrast, there was no overall difference in the auditory-evoked responses in naive mice (5 and 15 kHz; *p* = 0.52; Fig. 1*f*; Extended Data Fig. 1-1), suggesting the increased Ca^2+^ response to CS+ in tuft dendrites is due to learning-related plasticity of the auditory-evoked response. To further probe whether this increase in dendritic activity was indeed learning dependent and not simply because of stimuli exposure, we compared the average Ca^2+^ responses to both CS+ and CS– with a reference stimulus composed of a train of tones not previously presented to the mice (5 × 500 ms, 10 kHz; Extended Data Fig. 1-2). Here, the overall auditory-evoked Ca^2+^ response was significantly larger during CS+ (1.48 ± 0.05 ΔF/F*s; *n* = 52 dendrites, Fig. 1*g*) compared with either the CS– (1.12 ± 0.05 ΔF/F*s; *n* = 46 dendrites; *p* < 0.001; Fig. 1*g*) or the reference stimulus (1.33 ± 0.08 ΔF/F*s; *n* = 37 dendrites, *p* = 0.02; Fig. 1*g*). Importantly, there was no difference in the auditory-evoked Ca^2+^ response to CS– and the reference stimulus (*p* = 0.11; Fig. 1*g*), illustrating that the increase in dendritic activity in response to CS+ was learning dependent and not because of stimuli exposure.

**Figure 2.**
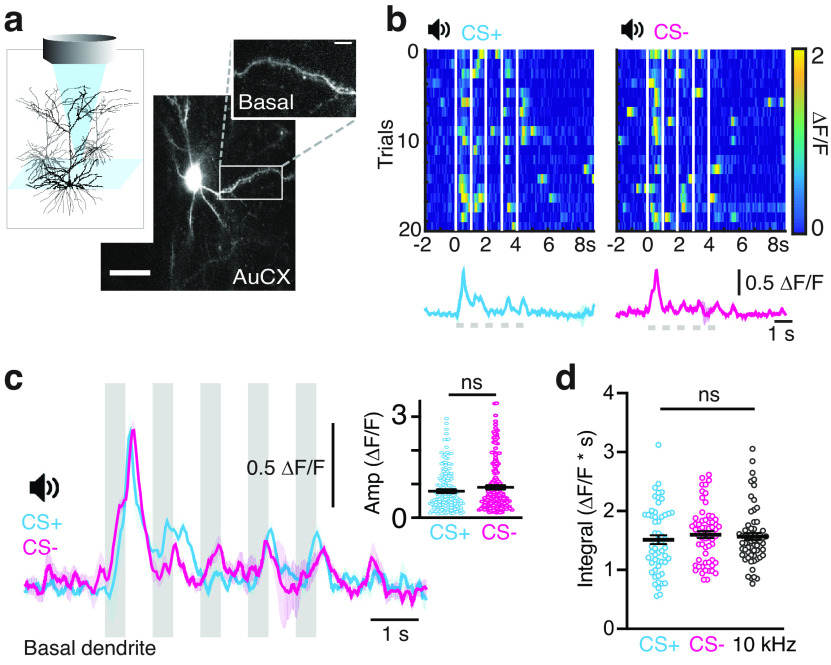
CS+ and CS– evoke similar responses in basal dendrites. ***a***, Top, Schematic of the experimental design. Example FOV of basal dendrites from L2/3 pyramidal neurons expressing GCaMP6f (located 100–250 μm below pia). Scale bar: 10 μm. Inset, Zoom image of basal dendrite. Scale bar: 5 μm. ***b***, Heatmaps of Ca^2+^ transients in response to CS+ (left) and CS– (right) in an example basal dendrite. White bars indicate timing of the tones (5 × 500 ms). Bottom, Trial-averaged Ca^2+^ responses to CS+ (cyan) and CS– (magenta). Bold line, Average response; light shading, SEM. ***c***, Overlay of the trial-averaged Ca^2+^ responses to CS+ (cyan) and CS– (magenta) from the same basal dendrite shown in ***b***. Gray bars show the tones in the auditory stimuli. Inset, Peak amplitude of the trial-averaged Ca^2+^ responses to CS+ (0.78 ± 0.04 ΔF/F) and CS– (0.90 ± 0.05 ΔF/F). Mann–Whitney test. ***d***, Integral of the trial-averaged ΔF/F responses to CS+ (CS+: 1.51 ± 0.07 ΔF/F*s), CS– (1.60 ± 0.05 ΔF/F*s), and a reference stimulus (10 kHz; 1.56 ± 0.05 ΔF/F*s; Extended Data Fig. 2-1). Mann–Whitney test. All data represented as mean ± SEM. ns: *p* > 0.05.

**Figure 3.**
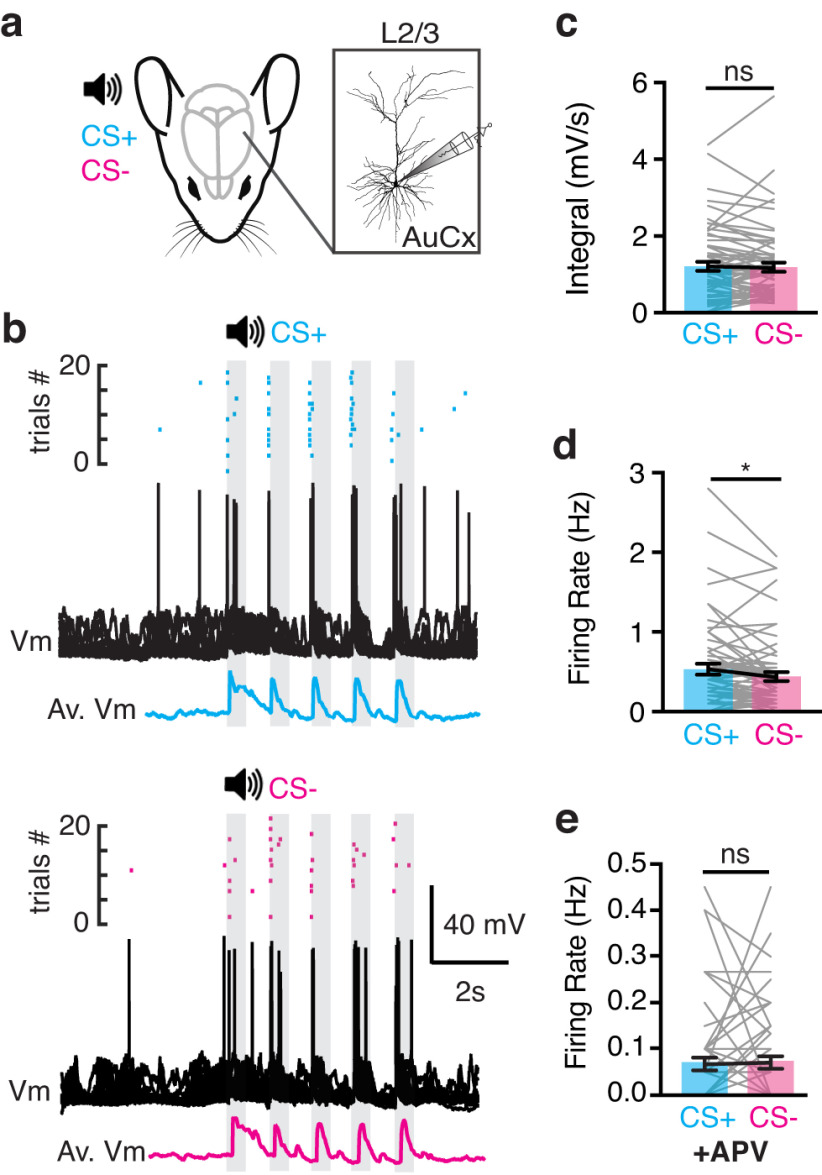
Action potential output is increased during CS+. ***a***, Schematic of the experimental design. In vivo patch clamp recordings were performed in the auditory cortex of urethane anaesthetized mice following auditory fear conditioning. ***b***, Raster plot of somatic action potentials (top), overlay of voltage responses (middle), and average subthreshold response (bottom) in an example L2/3 pyramidal neuron during CS+ (cyan) and CS– (magenta). ***c***, Integral of the subthreshold voltage response to each tone during CS+ (cyan) and CS– (magenta). Wilcoxon matched-pairs signed-rank test. ***d***, Average somatic firing rate during each tone in the CS+ (cyan) and CS– (magenta). Wilcoxon matched-pairs signed-rank test. ***e***, Average somatic firing rate during each tone in the CS+ (cyan) and CS– (magenta) after local cortical application of the NMDA channel agonist, APV. Wilcoxon matched-pairs signed-rank test. Bars represent mean ± SEM. ns: *p* > 0.05, * *p* < 0.05.

10.1523/ENEURO.0060-22.2022.f1-3 Extended Data Figure 1-3 Fear learning did not alter the correlation of Ca^2+^ transients across tuft dendrites of the same neuron. ***a***, 3D reconstruction (bottom) of a L2/3 pyramidal neurons (N1) from an example FOV (top). ROIs from the same neuron are highlighted in green. ***b***, Ca^2+^ transients recorded in tuft dendrites from the reconstructed neuron in ***a*** (Roi#10 and Roi#6; N1). Orange dots, Evoked local Ca^2+^ transients. ***c***, The correlation of Ca^2+^ responses in tuft dendrites from the same neuron is similar during CS+ (0.69 ± 0.07) and CS– (0.74 ± 0.06; *p* = 0.51). Mann–Whitney test. All values represent mean ± SEM. Download Figure 1-3, EPS file.

10.1523/ENEURO.0060-22.2022.f1-1 Extended Data Figure 1-1 The auditory stimuli used during fear conditioning evoked similar activity in the tuft dendrites of naive mice. ***a***, Two-photon calcium imaging was performed in tuft dendrites in response to the same stimuli used during fear condition-ing (5 × 500 ms, 5 or 15 kHz tones) in naive mice (*n* = 3) that did not experience the fear conditioning protocol. ***b***, Heatmaps of Ca^2+^ responses in an example tuft dendrite to both 5 and 15 kHz (top) and trial-averaged Ca^2+^ activity (bottom). ***c***, The peak amplitude of the trial-averaged Ca^2+^ response to the auditory stimuli was not significantly different (5 kHz, 0.73 ± 0.08 vs 15 kHz, 0.70 ± 0.08; *p* = 0.52; 3 mice; Mann–Whitney test). Download Figure 1-1, EPS file.

10.1523/ENEURO.0060-22.2022.f1-2 Extended Data Figure 1-2 Activity in tuft dendrites evoked by a reference stimulus (10 kHz). ***a***, Schematic of the experiment: dendritic recordings were performed from tuft dendrites of L2/3 pyramidal neurons in the auditory cortex. ***b***, Top, Heatmap of Ca^2+^ responses in an example tuft dendrite to a reference auditory stimulus (5 × 500 ms, 10 kHz). Bottom, Trial-averaged Ca^2+^ response from the same tuft dendrite. Download Figure 1-2, EPS file.

Is this learning-related plasticity encoded locally, on a single dendritic branch, or on multiple tuft dendrites within a single neuron? To address this, we reconstructed L2/3 pyramidal neurons *post hoc* and retrospectively assessed the auditory-evoked Ca^2+^ responses to conditioned stimuli in tuft dendrites from the same neurons (Extended Data Fig. 1-3; see Materials and Methods). Identifying branches from the same neuron illustrated that tuft dendrites typically had similar patterns of activity, however, auditory-evoked Ca^2+^ events were occasionally localized to a single branch. Following fear learning, the correlation of Ca^2+^ transients across tuft dendrites of the same neuron were similar during CS+ and CS– (Extended Data Fig. 1-3), suggesting that fear conditioning did not alter the overall dendritic pattern of activity. Taken together, we found a learning-related increase of auditory-evoked response in tuft dendrites of L2/3 pyramidal neurons, suggesting plasticity occurs within tuft dendrites following fear learning.

### CS+ and CS– evoke similar activity in basal dendrites

Does learning-related plasticity occur throughout an individual neuron, or is it compartmentalized to specific dendritic regions? To test this, we assessed whether fear learning also influences the processing of sensory information in the basal dendrites of L2/3 pyramidal neurons. Following the fear conditioning protocol, auditory-evoked Ca^2+^ activity was recorded in basal dendrites (>100 μm below pia; Fig. 2*a*,*b*). In contrast to tuft dendrites, CS+ and CS– evoked similar activity in basal dendrites, with no difference in the peak amplitudes (CS+: 0.78 ± 0.04; CS–: 0.90 ± 0.05, *n* = 56/61 dendrites, 8 mice, *p* = 0.25; Fig. 2*c*,*d*) and integral of the auditory-evoked Ca^2+^responses (CS+: 1.51 ± 0.07 ΔF/F*s; CS–: 1.60 ± 0.05 ΔF/F*s; *n* = 56/61 dendrites, 8 mice, *p* = 0.39; Fig. 2*d*). These findings suggest that the processing of sensory information in basal dendrites is not influenced by fear learning. To confirm that the auditory-evoked Ca^2+^ responses in basal dendrites was independent of stimuli exposure, we compared the Ca^2+^ response to both CS+ and CS– with a reference stimulus composed of a train of tones (5 × 500 ms, 10 kHz) not previously presented to the mice (10 kHz: 1.56 ± 0.05 ΔF/F*s; *n* = 64 dendrites; Extended Data Fig. 2-1). Here, there was no significant difference in the auditory-evoked Ca^2+^ responses of the reference stimulus compared with CS+ (*p* = 0.47) and CS– (*p* = 0.34; Fig. 2*e*). Furthermore, the average Ca^2+^ responses evoked by the reference stimulus were similar in both tuft and basal dendrites (*p* = 0.34; Extended Data Fig. 2-1), suggesting that neither the depth of recordings nor a spatially disparate influence of anesthesia contributed to the reported differences in the evoked Ca^2+^ activity in tuft and basal dendrites following fear learning. Taken together, these results suggest that, unlike in tuft dendrites, plasticity does not occur in the basal dendrites of L2/3 pyramidal neurons following fear learning.

10.1523/ENEURO.0060-22.2022.f2-1 Extended Data Figure 2-1 Activity in basal dendrites evoked by a reference stimulus (10 kHz). ***a***, Schematic of the experiment: dendritic recordings were performed from basal dendrites of L2/3 pyramidal neurons in the auditory cortex. ***b***, Top, Heatmap of Ca^2+^ responses in an example basal dendrite to a reference auditory stimulus (5 × 500 ms, 10 kHz). Bottom, Average Ca^2+^ response from the same basal dendrite. ***c***, Histogram distribution of the peak amplitude values of all the Ca^2+^ transients for tuft and basal dendrites evoked by the reference stimulus (10 kHz; all tones; *p* = 0.34 Mann–Whitney test). Download Figure 2-1, EPS file.

### Action potential output is increased following fear learning

Learning-related plasticity in tuft dendrites may increase the efficacy of distal inputs on the somatic output (Palmer et al., 2014). Therefore, we next assessed whether somatic activity is influenced by fear learning. To measure the somatic voltage response to CS+ and CS– auditory stimuli, whole-cell patch clamp recordings were performed from L2/3 pyramidal neurons in the auditory cortex of urethane anaesthetized mice following fear conditioning (Fig. 3*a*, *n* = 13 neurons, 10 mice; see Materials and Methods). Here, each tone within CS+ and CS– evoked a robust response consisting of a subthreshold voltage envelope and action potentials (Fig. 3*b*). Both CS+ and CS– evoked a similar subthreshold response (CS+, 1.20 ± 0.11 mV/s; CS–, 1.18 ± 0.11 mV/s; *n* = 13 neurons, 10 mice; *p* = 0.35; Fig. 3*c*). However, in contrast to the subthreshold response, the evoked firing rate was significantly greater during CS+ compared with CS– (CS+ 0.53 ± 0.06 Hz vs CS– 0.44 ± 0.05 Hz; *n* = 13 neurons, 10 mice; *p* = 0.01; Fig. 3*d*). These results illustrate that fear learning enhances somatic action potential output in the absence of detectable changes in the subthreshold voltage. This discrepancy suggests that the greater evoked firing rate during CS+ is not simply driven by the linear somatic summation of synaptic inputs, and may be because of dendritic electrogenesis which has previously been shown to directly generate action potentials without a measurable influence on subthreshold voltage (Larkum et al., 2009; Palmer et al., 2014). To test whether somatic output is influenced by modulating synaptic input in the upper cortical layers where distal dendrites reside, we locally applied the NMDA-receptor blocker APV onto the cortical surface while recording somatic voltage activity. Block of NMDA-dependent events in the upper cortical layers significantly dampened somatic activity and abolished the increased action potential rate following fear learning (CS+: 0.06 ± 0.01 Hz vs CS–: 0.07 ± 0.01 Hz; *p* = 0.73; Fig. 3*e*). These results suggest that fear learning enhances the somatic output of L2/3 pyramidal neurons within the auditory cortex, which is dependent on synaptic activation of NMDA channels in the upper cortical layers.

### Control of the somatic output by tuft dendrites

**Figure 4.**
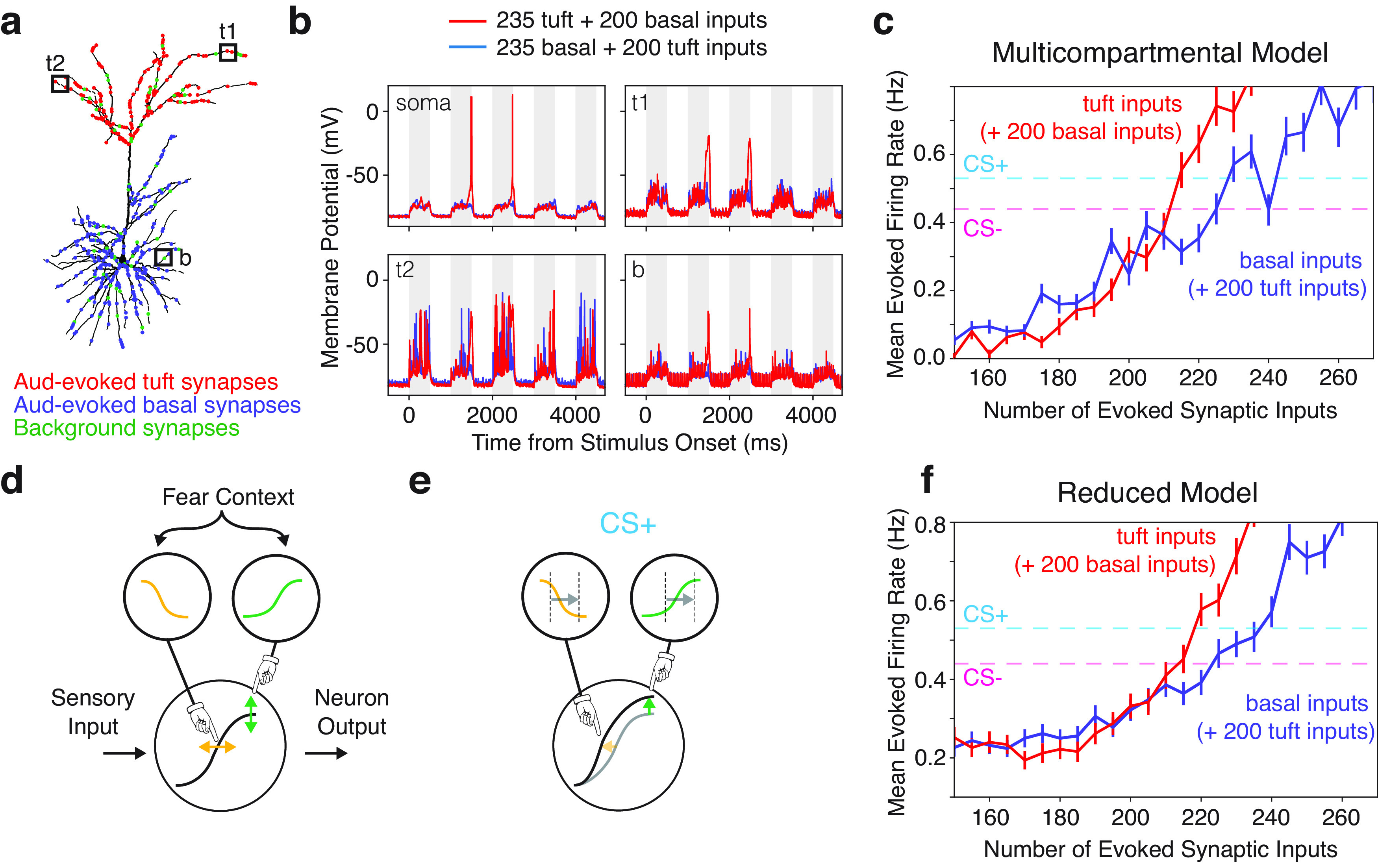
Control of the somatic output by tuft dendrites. ***a***, Schematic of the simulation design. A reconstructed L2/3 pyramidal neuron was used for a multicompartment model. Background synapses (green dots) were distributed uniformly across the neuron, while auditory-evoked synapses were distributed uniformly across the basal (blue dots) or tuft dendrites (red dots; see also Extended Data Fig. 4-1). ***b***, To simulate auditory-evoked responses in this L2/3 neuron, we put either 235 tuft and 200 basal (red traces), or 235 basal and 200 tuft (blue traces) auditory-evoked synapses during a 5 × 500 ms stimulation window. Example membrane potentials during these two simulation conditions are shown for two tuft locations (labels t1 and t2 in panel ***a***), the soma, and for one basal location (label b in panel ***a***). ***c***, To see how changes in tuft and basal inputs change the output of the neuron, we simulated an increasing number of auditory-evoked tuft inputs with a constant 200 basal auditory-evoked inputs (red) and an increasing number of auditory-evoked basal inputs with a constant 200 tuft auditory evoked-inputs (blue). Error bars are SEM. Dotted lines denote the experimentally determined mean firing rates for the CS+ (cyan) and CS– (magenta) conditions. ***d***, Schematic of the reduced model using two sigmoids to represent tuft inputs controlling the maximum (green) and threshold (orange) of the neuron input output function. ***e***, Compared with CS–, the neuron input output function has a decreased threshold (orange) and increased maximum (green) in response CS+ (illustrated by arrows). ***f***, The reduced model is able to recapitulate the simulation results from the multicompartmental model in ***c***).

Our experimental findings demonstrate that, following fear learning, auditory-evoked responses are enhanced in both the tuft dendrites and cell bodies of L2/3 pyramidal neurons. Does this increase in auditory-evoked signaling in tuft dendrites explain the changes in somatic firing rates following fear conditioning? To investigate this, we examined the influence of changes in synaptic input to tuft and basal dendrites on action potential generation using the NEURON simulation platform (see Materials and Methods; Fig. 4*a*; Extended Data Fig. 4-1). We modelled the relationship between the number of synaptic inputs to the tuft and basal dendrites that are required to generate the somatic action potential output recorded in the experimental data (Fig. 4*b*). During the auditory stimulus period, we simulated an increasing number of tuft (or basal) inputs with a constant 200 basal (or tuft) inputs. Overall, less synaptic inputs were required in tuft dendrites (210–215 synapses) compared with basal dendrites (220–245 synapses) to drive somatic action potential output that corresponded to the experimentally measured mean firing rate during CS– and CS+, respectively (Fig. 4*c*). These findings suggest that, given the nonlinear electrotonic nature of the tuft dendrites, not only is it plausible for changes in the tuft dendrites to account for our experimental results, tuft input can have a greater influence on somatic output compared with basal inputs, despite being further from the action potential initiation zone. To more intuitively understand how the nonlinearities in the pyramidal neurons can lead to changes in firing rates associated with fear conditioning we implemented a composite sigmoid model that explicitly represents the nonlinear interaction of the tuft and basal dendrites via two tuft sigmoids that control the maximum and threshold of the neurons input-output function (Fig. 4*d*). In this model, increasing excitatory input into the tuft dendrite decreases the threshold and increases the maximum value of the neurons input-output function in a nonlinear manner (Fig. 4*e*). Using this model, we simulated changes in tuft and basal inputs analogously to the simulations in the multicompartmental model. We found qualitatively similar results in this vastly simplified model (Fig. 4*f*), which in this case were easier to interpret. Increasing the basal inputs in both models resulted in a more linear response in output frequency compared with increasing tuft inputs. Furthermore, the results changing the basal versus tuft inputs diverged near the output firing rates associated with empirically measured CS– and CS+ conditions in our experiments. These results suggest that the L2/3 pyramidal neurons in our experiments could be taking advantage of the tuft nonlinearity to use a modest change in inputs to control the response to conditioned stimuli. Therefore, compartmentalized learning-related plasticity in tuft dendrites may provide a mechanism to increase the effect of distal feedback inputs on the somatic output, increasing the computational ability and flexibility of L2/3 pyramidal neurons.

## Discussion

The dendrites of pyramidal neurons dynamically encode sensory stimuli and perception (Xu et al., 2012; Palmer, 2014; Takahashi et al., 2016, 2020; Ranganathan et al., 2018) and are known to support different forms of plasticity (Holthoff et al., 2006; Makino and Malinow, 2011; Cichon and Gan, 2015; Brandalise et al., 2016; Sandler et al., 2016; Bittner et al., 2017; d'Aquin et al., 2022; O'Hare et al., 2022; Otor et al., 2022). Using two-photon Ca^2+^ imaging and patch clamp electrophysiology in L2/3 pyramidal neurons within the auditory cortex, we found that fear learning increases somatic output and enhances auditory-evoked Ca^2+^ responses in tuft, but not basal, dendrites. These results suggest fear learning enhances sensory processing in specific dendritic compartments, illustrating dendritic compartmentalization of learning-related plasticity.

The results in this study contribute to a growing body of evidence that shows the auditory cortex undergoes plasticity following auditory-fear learning (Romanski and LeDoux, 1992; Letzkus et al., 2011; Yang et al., 2016; Abs et al., 2018), where auditory-evoked activity in pyramidal neurons change both morphologically (Yang et al., 2016; Lai et al., 2018) and functionally (Weinberger and Diamond, 1987; Quirk et al., 1995; Letzkus et al., 2011; Gillet et al., 2018; Dalmay et al., 2019). Our findings also confirm the important role of cortical layer 1, where tuft dendrites reside, in driving these changes (Abs et al., 2018; Doron et al., 2020). Here, we demonstrate that auditory-evoked Ca^2+^ responses are specifically increased in apical tuft dendrites following fear learning. Such changes were not observed in basal dendrites, suggesting learning-related neuronal compartmentalization of dendritic plasticity. This dendrite-specific plasticity is not unique to cortical pyramidal neurons as recent studies have also reported compartmentalized plasticity in the lateral amygdala (d'Aquin et al., 2022) and hippocampus (O'Hare et al., 2022). Taken together, these results illustrate that neurons are multicompartmental (Häusser and Mel, 2003) which, by providing neurons with multiple independent integrative units that process information in parallel, increases the computational power of a single neuron (Poirazi et al., 2003; Jadi et al., 2014; Ujfalussy et al., 2018).

In addition to increasing the computational power of pyramidal neurons, compartmentalized experience-dependent plasticity may also provide a cellular mechanism to gate information received by a neuron, specifically enhancing the impact of certain input pathways. Since the tuft dendrites of pyramidal neurons are the target of long-range feedback projections from the fear pathway (Yang et al., 2016), the enhanced activity in tuft dendrites may act to ensure this synaptic pathway has a greater influence at the cell body. Indeed, we found experimental evidence that tuft dendrites strongly influence somatic output, increasing the effect of distal synaptic inputs on the cellular output. This was supported by two computational models of different complexity, which both showed that tuft inputs can have a greater influence on somatic output compared with basal inputs, despite being further from the action potential initiation zone. This may be a key mechanism to dynamically enhance top-down control over sensory encoding which is required during learning (Makino and Komiyama, 2015). The strong influence of tuft dendrites on somatic firing in our study is in agreement with other studies (Palmer et al., 2014; Goetz et al., 2021) showing that small changes in the synaptic inputs onto the distal dendrites of L2/3 pyramidal neuron can dynamically drive somatic activity (Goetz et al., 2021). This was further illustrated experimentally by pharmacologically modulating the synaptic input within layer 1 by blocking NMDA channels which decreased somatic action potentials. Together, our findings suggest that synaptic input onto basal and tuft dendrites may have different roles in contributing to the overall excitability of individual neurons. Since learning requires flexibility in sensory representation, plasticity within different dendritic compartments may dynamically shift the balance between feedback (tuft) and feedforward (basal) information onto a single neuron, resulting in flexile sensory encoding throughout learning (Jordan and Keller, 2020).

In this study, fear learning specifically caused an increase in the Ca^2+^ transient evoked in response to CS+ in tuft, and not basal, dendrites. The cause of this compartmentalized Ca^2+^ increase is unknown and could be because of both presynaptic and postsynaptic processes. For example, the increase in the tuft response following fear learning may be due to postsynaptic modification of synaptic weight, which can change the synaptic response to incoming input through long-term potentiation (Fusi and Abbott, 2007; Nabavi et al., 2014). Likewise, presynaptic modification of synaptic input from brain regions known to be essential for fear conditioning (LeDoux, 2000; Maren and Quirk, 2004) may also drive the increase in the tuft response to conditioned stimuli by altering their firing patterns and synaptic targets within the auditory cortex (Yang et al., 2016). Previous studies report that global Ca^2+^ signaling may result from backpropagating action potentials (Wilson et al., 2016; Iacaruso et al., 2017; Beaulieu-Laroche et al., 2018; Francioni et al., 2019; Kerlin et al., 2019). Although backpropagating action potentials may contribute to the dendritic Ca^2+^ signal recorded in our study, we suggest that they do not drive the compartmentalized increase in Ca^2+^ signaling within tuft dendrites as we would expect to see a similar change in the Ca^2+^ response in basal dendrites, which was not the case. Our findings further suggest that intracellular calcium stores also did not primarily drive the changes in dendritic Ca^2+^ responses, as fear learning did not alter the spatial pattern of dendritic activity which has recently been shown to be influenced by intracellular calcium release (O'Hare et al., 2022). Additional in-depth experiments are required to determine the presynaptic and/or postsynaptic modifications which drive the reported changes in dendritic signaling following fear learning, which is an exciting new avenue for future research.

It is important to note that the experiments reported in this study were performed under urethane anesthesia as, compared with other common anesthetic, urethane been shown to have a comparatively smaller influence on dendritic processing (Potez and Larkum, 2008). Although anesthesia reduces overall feedback activity in the cortex (Boly et al., 2011; Murphy et al., 2019), our findings illustrate that plasticity can still occur in the distal dendrites that are targeted by feedback input. Our results also suggest that the enhanced neural activity following fear learning is probably not because of arousal pathways or movement which are abolished during anesthesia (Salay et al., 2018; Bigelow et al., 2019; Stringer et al., 2019). Therefore, the differences in sensory processing following fear learning reported in this study illustrates the robust nature of the enhanced signaling as it was observed even when feedback pathways were dampened.

In summary, we identified compartmentalized learning-related plasticity in the processing of auditory stimuli in the tuft dendrites and somatic output of L2/3 pyramidal neurons. This is in direct contrast to the long held (and debated) hypothesis that, despite extensive dendritic arbors, neurons may function as a simple one-compartment model (Poirazi et al., 2003). The ability to adapt behavioral responses to the external environment relies on the flexibility of sensory representation that can be constantly updated through learning (Kato et al., 2015). Perhaps this is achieved through the increased cortical computation afforded by multiple independent integrative units, which may provide a cellular mechanism for the control of neural output during learning.

## Synthesis

Reviewing Editor: Arianna Maffei, Stony Brook University Decisions are customarily a result of the Reviewing Editor and the peer reviewers coming together and discussing their recommendations until a consensus is reached. When revisions are invited, a fact-based synthesis statement explaining their decision and outlining what is needed to prepare a revision will be listed below. The following reviewer(s) agreed to reveal their identity: NONE.

The reviewers agree that this study addresses an important question about how learning activated dendrite compartments. The major concerns raised regard methodological aspects and data interpretation. Some of the important methodological aspects include the ability to distinguish backpropagating signals from direct activation in the apical dendrites,the potential effect of urethane on masking or amplifying some effects of inhibition and the definition of ROI in the experiment/analysis.

Concerns about data interpretation include the link between dendrites activation and plasticity and perceived lack of coherence in the interpretation of data in Fig 1-3.

Detailed comments by the reviewers follow below.

Reviewer #1

The manuscript, “Dendritic compartmentalization of learning-related plasticity", examines whether learning related plastic changes in neuronal response is encoded within physically distinct subcellular compartments or rather globally across the entire neuron. By employing auditory fear conditioning as sensory learning that induces plasticity and two-photon calcium imaging and somatic patch clamp recording as the readout of plastic changes, the authors report that the fear conditioning indeed results in an increase in the somatic action potential response. Interestingly this learning causes enhancement of auditory-evoked Ca2+ responses in the apical tuft by not in the basal dendrites. Moreover, this enhanced response in the apical tuft was specific to the auditory cue that was paired with a footshock, and not to the unpaired or previously unexperienced cues. This study provides important evidence supporting the complexity of neuronal function which has long been described in multicompartment models that endows a neuron with an increased computational capacity. The manuscript is concisely written and easy to follow and will likely attract readership from a broad audience. However, there are a few key points which may bring the methodological rigor into question that I’d like to see addressed.

Major comments and concerns:

1. Ca2+ response analysis: It is my understanding that the “Ca2+ transients” analyzed in this study do not distinguish between local dendritic events and global backpropagating action potential associated transients except for what’s being indicated in Figure S3. Is that correct? This, I find to be problematic when interpreting the acquired data, especially when majority of the analyses seems to have been done on dendritic segments imaged in an upper layer without reconstructing the individual neurons (unless specifically mentioned as in Fig S3; see my comment #2). Is it possible that the apical tuft specific increase in peak amplitude is reflecting the increase in bAP associated Ca transients as predicted by the observed increase in the somatic firing rate? And this increase in bAPs is more impactful in the upper layer imaging of the dendritic tuft compared to the imaging of the basal dendrites in deeper layer 2/3 where photons are more prone to scattering and therefore images are overall dimmer as seen in Figure1f vs 2c?

2. ROI identification of layer 2/3 pyramidal neuron dendritic segments: It is unclear to me whether each dendritic ROIs, especially those in the apical tufts, were visually traced back to the soma to confirm their identity as layer 2/3 dendrites. While layer 2/3 basal dendrites would be more easily discernible, fluorescent apical dendrites as shown in Figs 1c and S3c likely contains layer 5 dendrites. Any contamination of layer 5 dendrites in apical tuft but not basal dendrites would likely introduce a confound in which increased bAP potentially seen in layer 5 dendrites will skew the average dendritic Ca2+ transient amplitudes imaged in the upper layer to increase (30-80um; “apical tuft”) but no in the basal dendrites. I would appreciate more information on the ROI identification process, specifically focusing on the criteria to avoid layer 5 dendrite contamination.

3. How to reconcile interpretations of Figs 1f, 2c, Fig3d and S3c: I am having bit of a trouble reconciling the findings shown in these figures. Figs 1 and 2 together indicates that there is an apical tuft dendrite-specific increase in Ca2+ transients in response to auditory conditioning (albeit the difference is very little). Fig3 then shows that somatically recorded AP response is indeed also increased (increased firing rate). So these together may imply that an increase in the active dendritic event in the apical tuft promotes axonal spiking. But then, this increase in bAPs is not “seen” by the basal dendrites. Unless the authors are actively subtracting all bAPs from their Ca2+ transients, I don’t understand how this is possible. In addition, data in FigS3 indicates that the increased tuft Ca2+ amplitudes do not change the correlation coefficients. How does one interpret this result? The simplest prediction would be as follows: If instances of local dendritic spikes were increased due to plasticity strengthening the distal feedback inputs, then the coefficients would decrease. If there is an increase in spiking activity (and if indeed bAPs are NOT subtracted from the Ca2+ transients), then the coefficients may increase, or may stay the same if global dendritic arbor-wide transients dominate the signals. In the current manuscript, no interpretations or significance of the finding is offered. Can authors discuss potential implication of this result in discussion?

Minor comments

1. Stats: It appears that only behavioral analysis (Fig 1b) and somatic recording analyses (Fig 3) were performed as paired tests. Why is it that dendritic Ca2+ transients weren’t analyzed as paired tests?

2. Figure S3: At least on my computer, it doesn’t look like the color-coding schemes matches between the top and bottom images of Figure S3a.

3. Urethane anesthesia: Authors explain that their choice of urethane anesthesia “limits the potentially confounding effect of state dependent modulation of brain activity”. Have the authors observe any specific effects of the increased up and down states induced by urethane?

Reviewer #2

This manuscript describes a series of interesting experiments describing how tuft synapses/dendrites and basal synapses/dendrites regulate cell firing differently, and how they change their responsiveness to auditory input differently following auditory fear conditioning. In an excellent mixture of approaches, both in vivo calcium imaging and patch-clamping, and Neuron modelling techniques were used. This work advances our understanding of auditory cortex information processing, although there are some methodology and interpretation questions that need to be addressed.

Major comments

1. Methodologically, the experiments were conducted in urethane anesthetized animals, as the authors duly note and discuss. I believe that more thought could be put into this issue though. For example, does the altered inhibitory control, which presumably accompanies urethane, act in a way that disconnects communication between the two sets of dendrites, or for example the ability to generate back-propagating APs? There may be other related issues that could deepen the consideration of this matter.

2. It appears from Fig S5 that there are no channels on the modeled apical dendrite. Is that correct? Won’t that relate to the issue above, and affect the communication throughout the cell and the ability to generate bAPs? Or is the soma-apical dendrite one compartment? Is that realistic? I also don’t understand why there are virtually no channels in the basal compartment. This would definitely affect whether there would be linear vs non-linear input-output relations when adding more synapses.

3. My main comment is that I don’t think it is possible to say that fear conditioning has caused plasticity in the tuft compartment, just because the calcium response has changed. It’s interesting that this tuft compartment gets top-down input. Therefore, it is entirely possible that the plasticity occurs somewhere else in the cortex or other input region and that this enhanced response is merely due to enhanced input coming from elsewhere. A constant input pre- and post-conditioning is needed to assess whether plasticity is occurring at the tufts of these cells. The auditory stimulus alone is not sufficient to ensure this, particularly at the top-down synapses. The result is still interesting and important, but the evidence for claimed plasticity itself is not there I feel.

4. If there is increased cell firing after fear conditioning, I wonder why there is not an increased calcium signal in the basal dendrites through bAPs. Are there not VGCCs in those dendrites?

Minor comments

1. It would be good if all the model conductance were defined.

2. The modeled cell doesn’t appear to have sAHPs. Is that the case in the patched cells? It’s hard to tell from the traces.

3. More details are needed in the Methods on the statistical tests used and the version of Prism that was used to do them

## Author Response

Reply to Reviewers

Computational Neuroscience Model Code Accessibility Comments for Author (Required):

N/A

Significance Statement Comments for Author (Required):

The topic under study has long been theorized, but experimental evidence in vivo has so far been lacking. There is agreement that results from this study provide an important contribution as they show distinct patterns of basal and apical dendrites activation during a learning task.

Comments on the Visual Abstract for Author (Required):

The visual abstract is informative and well designed

Synthesis Statement for Author (Required):

The reviewers agree that this study addresses an important question about how learning activated dendrite compartments. The major concerns raised regard methodological aspects and data interpretation. Some of the important methodological aspects include the ability to distinguish backpropagating signals from direct activation in the apical dendrites, the potential effect of urethane on masking or amplifying some effects of inhibition and the definition of ROI in the experiment/analysis.

Concerns about data interpretation include the link between dendrites activation and plasticity and perceived lack of coherence in the interpretation of data in Fig 1-3.

We thank the reviewers for their helpful suggestions and we address all comments below.

Detailed comments by the reviewers follow below.

Reviewer #1

The manuscript, “Dendritic compartmentalization of learning-related plasticity", examines whether learning related plastic changes in neuronal response is encoded within physically distinct subcellular compartments or rather globally across the entire neuron. By employing auditory fear conditioning as sensory learning that induces plasticity and two-photon calcium imaging and somatic patch clamp recording as the readout of plastic changes, the authors report that the fear conditioning indeed results in an increase in the somatic action potential response. Interestingly this learning causes enhancement of auditory-evoked Ca2+ responses in the apical tuft by not in the basal dendrites. Moreover, this enhanced response in the apical tuft was specific to the auditory cue that was paired with a footshock, and not to the unpaired or previously unexperienced cues. This study provides important evidence supporting the complexity of neuronal function which has long been described in multicompartment models that endows a neuron with an increased computational capacity. The manuscript is concisely written and easy to follow and will likely attract readership from a broad audience. However, there are a few key points which may bring the methodological rigor into question that I’d like to see addressed.

We thank the reviewer for their appreciation of the study and helpful comments on the manuscript.

Major comments and concerns:

1. Ca2+ response analysis: It is my understanding that the “Ca2+ transients” analyzed in this study do not distinguish between local dendritic events and global backpropagating action potential associated transients except for what’s being indicated in Figure S3. Is that correct? This, I find to be problematic when interpreting the acquired data, especially when majority of the analyses seems to have been done on dendritic segments imaged in an upper layer without reconstructing the individual neurons (unless specifically mentioned as in Fig S3; see my comment #2). Is it possible that the apical tuft specific increase in peak amplitude is reflecting the increase in bAP associated Ca transients as predicted by the observed increase in the somatic firing rate? And this increase in bAPs is more impactful in the upper layer imaging of the dendritic tuft compared to the imaging of the basal dendrites in deeper layer 2/3 where photons are more prone to scattering and therefore images are overall dimmer as seen in Figure1f vs 2c?

In this study, we did not distinguish between local and global events for all neurons. We did, however, identify local dendritic events in a subset of reconstructed neurons (Figure S3 / Extended Data Figure 1-3). While we agree that this distinction is important, we do not believe that the specific increase in the peak amplitude of evoked Ca2+ transients in the apical tuft following fear learning is simply reflecting the increased somatic firing rate as 1) an increase in Ca2+ transient amplitude did not occur in basal dendrites, despite backpropagating action potentials attenuating less in basal than apical dendrites, 2) the backpropagation of action potentials into tuft dendrites of L2/3 pyramidal neurons is reported as being mostly attenuated and unreliable (Waters et al 2003), and 3) the firing rate was decreased when the NMDA channels in the upper cortical layers were blocked. We now discuss this in the revised manuscript.

In the revised manuscript, we have now added a comparison of auditory-evoked calcium transients in the tuft and basal dendrites in response to a control auditory stimulus (10 kHz, naïve). As seen in new Figure S4 / Extended Data Figure 2-1, the average amplitude of the auditory-evoked response in the tuft and basal dendrites were not significantly different (p=0.34; Figure S4c / Extended Data Figure 2-1c). These results illustrate that increased scattering with imaging depth does not confound the results, and we now discuss this in the revised manuscript.

2. ROI identification of layer 2/3 pyramidal neuron dendritic segments: It is unclear to me whether each dendritic ROIs, especially those in the apical tufts, were visually traced back to the soma to confirm their identity as layer 2/3 dendrites. While layer 2/3 basal dendrites would be more easily discernible, fluorescent apical dendrites as shown in Figs 1c and S3c likely contains layer 5 dendrites. Any contamination of layer 5 dendrites in apical tuft but not basal dendrites would likely introduce a confound in which increased bAP potentially seen in layer 5 dendrites will skew the average dendritic Ca2+ transient amplitudes imaged in the upper layer to increase (30-80um; “apical tuft”) but no in the basal dendrites. I would appreciate more information on the ROI identification process, specifically focusing on the criteria to avoid layer 5 dendrite contamination.

In this study, we used sparse labelling of neurons to ensure that we were able to visually confirm the identity of all recorded neurons. This involved visually following tuft dendrites to the soma of origin during all imaging sessions. We also acquired z-stacks for all recording sessions and reconstructed neurons within the field of view in a subset of experiments (Figure S3 / Extended Data Figure 1-3). We now include greater information regarding the ROI identification process and confirmation of neuron identity in the Methods of the revised manuscript.

3. How to reconcile interpretations of Figs 1f, 2c, Fig3d and S3c: I am having bit of a trouble reconciling the findings shown in these figures. Figs 1 and 2 together indicates that there is an apical tuft dendrite-specific increase in Ca2+ transients in response to auditory conditioning (albeit the difference is very little). Fig3 then shows that somatically recorded AP response is indeed also increased (increased firing rate). So these together may imply that an increase in the active dendritic event in the apical tuft promotes axonal spiking. But then, this increase in bAPs is not “seen” by the basal dendrites. Unless the authors are actively subtracting all bAPs from their Ca2+ transients, I don’t understand how this is possible. In addition, data in FigS3 indicates that the increased tuft Ca2+ amplitudes do not change the correlation coefficients. How does one interpret this result? The simplest prediction would be as follows: If instances of local dendritic spikes were increased due to plasticity strengthening the distal feedback inputs, then the coefficients would decrease. If there is an increase in spiking activity (and if indeed bAPs are NOT subtracted from the Ca2+ transients), then the coefficients may increase, or may stay the same if global dendritic arbor-wide transients dominate the signals. In the current manuscript, no interpretations or significance of the finding is offered. Can authors discuss potential implication of this result in discussion?

The reviewer is correct - overall, our study illustrates there is an increased response in the firing rate and calcium activity in tuft, but not basal, dendrites following fear learning. These results are in agreement with previous studies which illustrate tuft dendrites can have a dramatic influence of somatic firing (Goetz et al, 2021), and bAP backpropagation into basal dendrites does not necessarily drive calcium responses in vivo (Palmer et al, 2014). Therefore, our results show that fear learning causes changes to calcium responses in tuft dendrites which can drive changes in somatic firing (please note, we do not subtract bAPs from our calcium transients). These conclusions were also confirmed in two computational models, which illustrated that indeed, learning-related increases in tuft dendrites can account for the changes in somatic output. Different effects on apical and basal dendrites activity following synaptic plasticity have also been recently reported (O’Hare et al, 2022). We now include greater discussion of this in the revised manuscript.

Our findings suggest that fear learning does not change the number of local events as the correlation of calcium activity across tuft dendrites of the same neuron does not change following fear learning (Figure S3 / Extended Data Figure 1-3). We now include greater discussion of this in the revised manuscript.

Minor comments

1. Stats: It appears that only behavioral analysis (Fig 1b) and somatic recording analyses (Fig 3) were performed as paired tests. Why is it that dendritic Ca2+ transients weren’t analyzed as paired tests?

Since calcium transients were not always evoked in response to both auditory stimuli tested, we were unable to do paired analysis on the dendritic calcium data.

2. Figure S3: At least on my computer, it doesn’t look like the color-coding schemes matches between the top and bottom images of Figure S3a.

We thank the reviewer for pointing this out. To avoid confusion, we now only show the example reconstructed neuron corresponding to the calcium traces shown in Figure S3b / Extended Data Figure 1-3b.

3. Urethane anesthesia: Authors explain that their choice of urethane anesthesia “limits the potentially confounding effect of state dependent modulation of brain activity”. Have the authors observe any specific effects of the increased up and down states induced by urethane?

We did not observe any reportable influence of fear learning on up and down states, nor on the subthreshold auditory-evoked response (see Figure 3c). Urethane was the choice of anaesthetics in this study because it has been shown to have the ‘least’ effect on dendritic and somatic activity compared to other common anaesthetics (Potez and Larkum, 2007). We now discuss this in the revised manuscript.

Reviewer #2

This manuscript describes a series of interesting experiments describing how tuft synapses/dendrites and basal synapses/dendrites regulate cell firing differently, and how they change their responsiveness to auditory input differently following auditory fear conditioning. In an excellent mixture of approaches, both in vivo calcium imaging and patch-clamping, and Neuron modelling techniques were used. This work advances our understanding of auditory cortex information processing, although there are some methodology and interpretation questions that need to be addressed.

We thank the reviewer for their helpful comments and suggestions.

Major comments

1. Methodologically, the experiments were conducted in urethane anesthetized animals, as the authors duly note and discuss. I believe that more thought could be put into this issue though. For example, does the altered inhibitory control, which presumably accompanies urethane, act in a way that disconnects communication between the two sets of dendrites, or for example the ability to generate back-propagating APs? There may be other related issues that could deepen the consideration of this matter.

Although we agree that it is possible that anaesthesia may influence tuft and basal dendrites differently, this would not explain our findings. Firstly, it is reasonable to expect that both CS+ and CS- should be equally influenced by anaesthesia, however our results show a specific change in tuft dendritic responses to CS+ only. Secondly, auditory-evoked activity was similar when comparing tuft and basal dendritic Ca2+ responses to a neutral stimulus (10kHz; new Figure S4 / Extended Data Figure 2-1), suggesting anaesthesia does not have a different influence on the different dendritic compartments. We now discuss this in the revised manuscript.

2. It appears from Fig S5 that there are no channels on the modeled apical dendrite. Is that correct? Won’t that relate to the issue above, and affect the communication throughout the cell and the ability to generate bAPs? Or is the soma-apical dendrite one compartment? Is that realistic? I also don’t understand why there are virtually no channels in the basal compartment. This would definitely affect whether there would be linear vs non-linear input-output relations when adding more synapses.

As stated in the Methods, ‘intrinsic membrane mechanisms were taken from the Hay et. al, (2011) layer 5b pyramidal model (Hay et al., 2011) and consisted of ten active conductances, internal Ca2+ dynamics, and passive conductances’. Both apical and basal had voltage dependent NMDA and AMPA channels. The apical dendrites in the model also contained Ca LVA, Ca HVA, SKv3, SKE2 channels (see Figure S5c / Extended Data Figure 4-1c). Although the basal dendrites did not contain these active conductances, the backpropagation of action potentials into basal dendrites within the model were similar to direct measurements (Nevian et al, 2007). Furthermore, similar distributions of active and passive conductances are commonly used in the literature as they reliably replicate patterns of activity recorded from direct electrophysiological recordings.

3. My main comment is that I don’t think it is possible to say that fear conditioning has caused plasticity in the tuft compartment, just because the calcium response has changed. It’s interesting that this tuft compartment gets top-down input. Therefore, it is entirely possible that the plasticity occurs somewhere else in the cortex or other input region and that this enhanced response is merely due to enhanced input coming from elsewhere. A constant input pre- and post-conditioning is needed to assess whether plasticity is occurring at the tufts of these cells. The auditory stimulus alone is not sufficient to ensure this, particularly at the top-down synapses. The result is still interesting and important, but the evidence for claimed plasticity itself is not there I feel.

We now include greater discussion in the revised manuscript and highlight the potential different sources of plasticity. Interestingly, a recent paper has shown compartmentalised plasticity in the dendrites of lateral amygdala neurons during fear conditioning (d’Acquin et al., 2022). We now include this reference in the revised manuscript.

4. If there is increased cell firing after fear conditioning, I wonder why there is not an increased calcium signal in the basal dendrites through bAPs. Are there not VGCCs in those dendrites?

As previously reported (Palmer et al, 2014), there is not always a high correlation between somatic output and calcium activity in basal dendrites. This result is indeed interesting and although out to the scope of this study, this finding presents an exciting avenue for future research.

Minor comments

1. It would be good if all the model conductance were defined.

All the information on all conductances used in the model are included in Figure S5c / Extended Data Figure 4-1.

2. The modeled cell doesn’t appear to have sAHPs. Is that the case in the patched cells? It’s hard to tell from the traces.

Both the modelled and patched cells have the ‘normal’ kinetics of an action potential as recorded in vivo. We now include a single action potential in Figure S5e / Extended Data Figure 4-1e.

3. More details are needed in the Methods on the statistical tests used and the version of Prism that was used to do them

We now include all details regarding Prism and the statistical tests used in the revised manuscript.
